# Relationship of Gensini score with retinal vessel diameter and arteriovenous ratio in senile CHD

**DOI:** 10.1515/biol-2021-0068

**Published:** 2021-07-16

**Authors:** Ning Wang, Changsen Liang

**Affiliations:** Department of Cardiovascularology, Jinan Seventh People’s Hospital, Jinan 250132, Shandong Province, China; Department of Ophthalmology, Jinan Seventh People’s Hospital, 21th Gongye North Road, Licheng District, Jinan 250132, Shandong Province, China

**Keywords:** CHD in the elderly, Gensini score, retinal vessel diameter, correlation

## Abstract

**Background:**

This study aimed to find the correlation of Gensini score with retinal vessel diameter and arteriovenous ratio in elderly patients with coronary heart disease (CHD).

**Methods:**

This study included 120 senile CHD patients as the CHD group and 100 healthy individuals as the normal group (NG). Gensini score was used to evaluate the severity of coronary artery lesions. Central retinal artery equivalents (CRAE), central retinal venular equivalents (CRVE), and arteriovenous ratio (AVR) were measured.

**Results:**

CHD group has lower CRAE and AVR than NG, while higher CRVE was observed in NG. CRAE and AVR in UAP (unstable angina pectoris) and AMI (acute myocardial infarction) groups showed reduction (stable angina pectoris); however, enhanced CRVE and Gensini scores in UA and AMI groups were observed as compared to the SAP group. CRAE and AVR in moderate and severe groups were reduced to a greater extent compared to the mild groups, while enhanced CRVE and Gensini scores were observed more often in the severe group than the mild group. CRAE and AVR were negatively correlated with the Gensini score; however, CRVE was positively correlated with the Gensini score.

**Conclusion:**

AVR is expected to be a noninvasive index to diagnose and predict senile CHD, which has a certain evaluation value. Diabetes, smoking history, and TC are independent risk factors of senile CHD.

## 1 Introduction

Coronary heart disease (CHD) is a familiar cardiovascular disease and chronic disease in the elderly [1]. With the aging of the population and the lifestyle changes, the incidence and mortality of CHD are increasing [2]. Related studies show that CHD incidence increases with age, and a high incidence of CHD is observed in the elderly [3]. The primary pathological basis of CHD is atherosclerosis [4]. Risk factors of CHD include age, genetic factors, hypertension, diabetes, dyslipidemia, overweight, obesity, and smoking. Clinical diagnosis is mainly performed by coronary CT, coronary angiography, and other imaging examinations [5]. Previous studies have reported that senile CHD patients have severe coronary artery stenosis and poor prognosis, which has seriously endangered the physical and mental health of senile CHD patients and reduced their quality of life [6]. Gensini score is an effective index to test the severity of the lesion and can accurately test the patient’s condition [7]. In the past, many studies explored the methods of predicting and evaluating the lesions, mainly including the determination of many biomarkers such as vascular-related inflammatory factors, growth differentiation factors, and angiopoietin-like proteins in the body, and imaging techniques such as coronary angiography [8,9,10]. However, there are some disadvantages such as high cost and trauma to the body of these methods [11]. Therefore, it is of great clinical significance to find a simple, noninvasive, convenient, economic, and repeatable detection index for an early and accurate evaluation of the degree of lesions to prevent senile CHD.

Many blood vessels in a typical organism have certain similarities in hemodynamic effects and metabolic environment, so the possibilities of developing certain types of diseases in these blood vessels are also similar [12,13]. More and more studies show that atherosclerosis is a systemic arterial disease, and microangiopathy acts in the pathogenesis of CHD [14,15]. Retinal vessels are the only vessels that can be directly observed by noninvasive methods, which provides a good option to test the microvascular function of the whole body [16,17]. As retinal vessels and coronary arteries have similar anatomical and physiological characteristics, the change of their diameters may indicate structural damage or functional changes, which has a particular early warning effect on CHD [18]. Color fundus photography allows to observe the pathological changes of retinal vessels and their surrounding tissues through the pupil, quantitatively analyze the diameter of retinal blood vessels with the aid of auxiliary software, record and save imaging data, facilitate systematic observation and analysis, and have the advantages of noninvasive safety, reliable information, simplicity, and low cost, which can provide important information for observing and studying the structure and functional states of body blood vessels [19,20].

In this study, the authors have taken color fundus photography for senile CHD patients, directly measured the diameter of retinal artery and vein with the help of computer technology, calculated the ratio of artery and vein, and discussed their correlation with Gensini score.

## 2 Materials and methods

### 2.1 General data

Altogether 120 senile CHD patients from January 2017 to June 2019 were selected as the CHD group, including 72 men and 48 women, aged 60–72 years, with an average of 65.25 ± 4.92 years. Inclusion criteria of this study were as follows: the diagnosis conformed to the diagnostic criteria of CHD formulated by WHO; the lesions were determined by coronary angiography, which showed that the primary branch stenosis was ≥50% or the secondary branch stenosis was ≥75%; and the lesion severity was determined by the Gensini method. According to different clinical types, patients with CHD were grouped into SAP, UA, and AMI. According to the Gensini score, patients with CHD were grouped into mild with 1–30 points, moderate with 31–60 points, and severe with 60 points. Exclusion criteria of this study were as follows: patients with eye refractive system diseases cannot be examined with ocular fundus; patients with congenital ocular vascular diseases; patients with a malignant tumor, autoimmune disease, infectious disease, and severe organ dysfunction; patients with acute and chronic infection; patients with mental illness; and patients without complete clinical data. Besides this, 100 healthy people in our hospital were selected as the normal group (NG), including 58 men and 42 women, aged 60–70 years, with an average of (65.58 ± 5.02) years.


**Informed consent:** Informed consent has been obtained from all individuals included in this study.
**Ethical approval:** The research related to human use has been complied with all the relevant national regulations, institutional policies, and in accordance with the tenets of the Helsinki Declaration, and has been approved by the authors’ institutional review board or equivalent committee.

### 2.2 Examination method

#### 2.2.1 Coronary angiography and assessment of the degree of vascular lesion

All patients with CHD were examined by coronary angiography using Philips CV Integris angiography machine with standard right femoral artery approach or right radial artery approach. Each lesion was determined by more than two orthogonal project positions, and the stenosis degree was expressed by the percentage of coronary artery diameter stenosis. Two cardiovascular interventional physicians determined the results. According to the location and degree of the lesion, the score was calculated by Gensini score standard to test the extent and severity of ischemia caused by the lesion.

Gensini score evaluation criteria [21] were as follows: Gensini score is the sum of coronary stenosis degree score and lesion site score. Stenosis degree score was as follows: the stenosis degree between 1 and 25% was recorded as 1 point; 26–50% as 2 points; 51–75% as 4 points; 76–90% as 8 points; 91–99% as 16 points; and 100% as 32 points. The lesion score is the product of a single lesion score and coefficient: the coefficient indicates the importance of stenosis in different positions of the coronary artery system, and the coefficients of each site are as follows: left trunk: 5, proximal left anterior descending branch: 2.5, middle left anterior descending branch: 1.5, aorta and first diagonal branch: 1, second diagonal branch: 0.5, distal left anterior descending branch: 1; the proximal left circumflex branch: 2.5, the middle left circumflex branch: 2.5, the distal left circumflex branch: 1, the blunt edge branch: 0.5; the proximal segment of a right coronary artery: 1, the middle segment of a right coronary artery: 1, the distal segment of a right coronary artery: 1, posterior descending branch: 1, the posterior branch of the left ventricle: 0.5. Gensini score with 1–30 points was regarded as mild; 31–60 as moderate; and >60 as severe.

#### 2.2.2 Measurement of retinal vessel diameter

All participants were examined by fundus photography: compound tropicamide eye drops were given to mydriasis, one drop each time, with an interval of 15 min, and the eyes were dropped twice in total, and fundus photography was performed 30 min later. The steps were performed by an experienced ophthalmologist using a Topcon fundus camera in Japan in a dark room, and a 45° elevation angle of both eyes centered on the fovea of optic disc and macula was selected, and the retinal images of both eyes were obtained and stored. The retinal vessel diameter was measured by IVAN software (University of Wisconsin, Madison): in the area of 0.5–1 DD away from the edge of the optic disc in color fundus photographs, the blood vessel diameters of six large retinal artery and vein branches were automatically identified and measured by the software. Par–Hubbard–Knudtson correction formula was applied to calculate the central retinal artery equivalent (CRAE), central retinal vein equivalent (CRVE), and the arteriovenous ratio (AVR) = CRAE/CRVE.

#### 2.2.3 Blood biochemical indicators

A fasting venous blood sample was obtained after 12 h on an empty stomach, and blood lipids were detected by Roche modular automatic biochemical analyzer, along with total cholesterol (TC), triglycerides (TGs), high-density lipoprotein cholesterol (HDL-C), and low-density lipoprotein cholesterol (LDL-C).

### 2.3 Statistical method

SPSS 20.0 was applied for the statistical analysis, and Graph Pad Prism 6 for visualizing the figures. The measurement data was represented by mean ± SD and compared by independent sample *t*-test. The comparison before and after treatment was made by paired *t*-test. The multiple comparisons were made by one-way ANOVA, and the pairwise comparison among groups was made by the SNK-q test. Counting data were represented by several cases/percentage [*n* (%)] and compared by the Chi-square test. When the theoretical frequency in the Chi-square test was less than 5, the continuity correction Chi-square test was applied. ROC was applied to test the diagnostic value of AVR in patients with CHD. Pearson correlation coefficient was applied to analyze the correlation of Gensini score with CRAE, CRVE, and AVR. Multivariate logistic regression was applied to analyze the risk factors affecting the severity of the lesions in patients with CHD. *P* < 0.05 indicated statistical difference.

## 3 Results

### 3.1 General data

There was no evident difference in sex, age, history of hypertension, diabetes, drinking, smoking, TC, TG, HDL-C, and LDL-C between the CHD and NG (*P* > 0.05; Table 1).

**Table 1:** Comparison of general data between the CHD group and the NG [*n* (%), mean ± SD]

Category	*n*	NG (*n* = 100)	CHD group (*n* = 120)	*χ*2/t	*P*
**Gender**				0.090	0.763
Male	130	58 (58.00)	72 (60.00)		
Female	90	42 (42.00)	48 (40.00)		
**Age (years)**	220	65.58 ± 5.02	65.25 ± 4.92	0.490	0.624
**History of hypertension**				0.119	0.730
No	116	54 (54.00)	62 (51.67)		
Yes	104	46 (46.00)	58 (48.33)		
**History of diabetes**				0.048	0.825
No	160	72 (72.00)	88 (73.33)		
Yes	60	28 (28.00)	32 (26.67)		
**Drinking history**				0.055	0.814
No	170	78 (78.00)	92 (76.67)		
Yes	50	22 (22.00)	28 (23.33)		
**Smoking history**				0.061	0.804
No	119	55 (55.00)	64 (53.33)		
Yes	101	45 (45.00)	56 (46.67)		
**Gensini score (Points)**					
1–30	40	—	40 (33.33)		
31–60	45	—	45 (37.50)		
>60	35	—	35 (29.17)		
**TC (mmol/L)**	220	4.50 ± 0.40	4.56 ± 0.42	1.078	0.282
**TG (mmol/L)**	220	1.34 ± 0.21	1.38 ± 0.27	1.208	0.228
**HDL-C (mmol/L)**	220	1.15 ± 0.16	1.18 ± 0.17	1.338	0.182
**LDL-C (mmol/L)**	220	3.02 ± 0.32	3.09 ± 0.36	1.510	0.132

### 3.2 Comparison of CRAE, CRVE, and AVR between CHD group and NG

**Figure 1:**
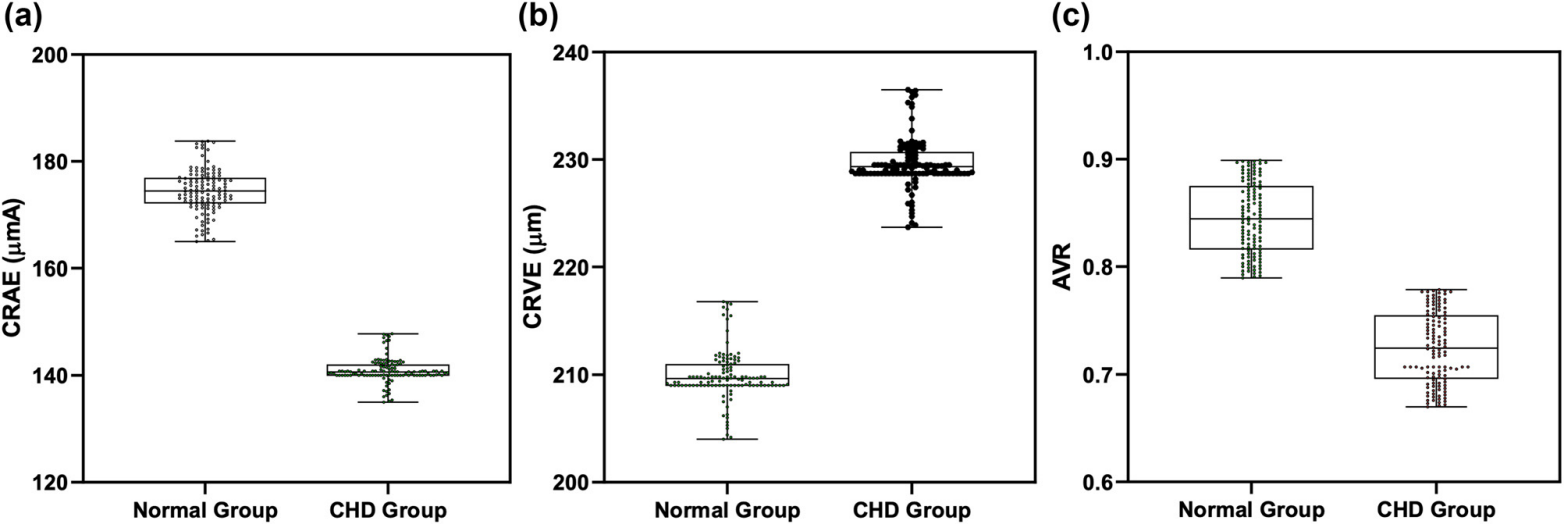
Comparison of CRAE, CRVE, and AVR between CHD group and NG. (a) CRAE in CHD group was reduced than that in NG. (b) CRVE in CHD group was larger than that in NG. (c) AVR in CHD group was reduced than that in NG. Note: ***P < 0.001.

CRAE, CRVE, and AVR in the CHD group were reduced compared to those in the NG group (*P* < 0.05; Figure 1).

### 3.3 Comparison of CRAE, CRVE, AVR, and Gensini scores in different CHD groups

**Figure 2:**
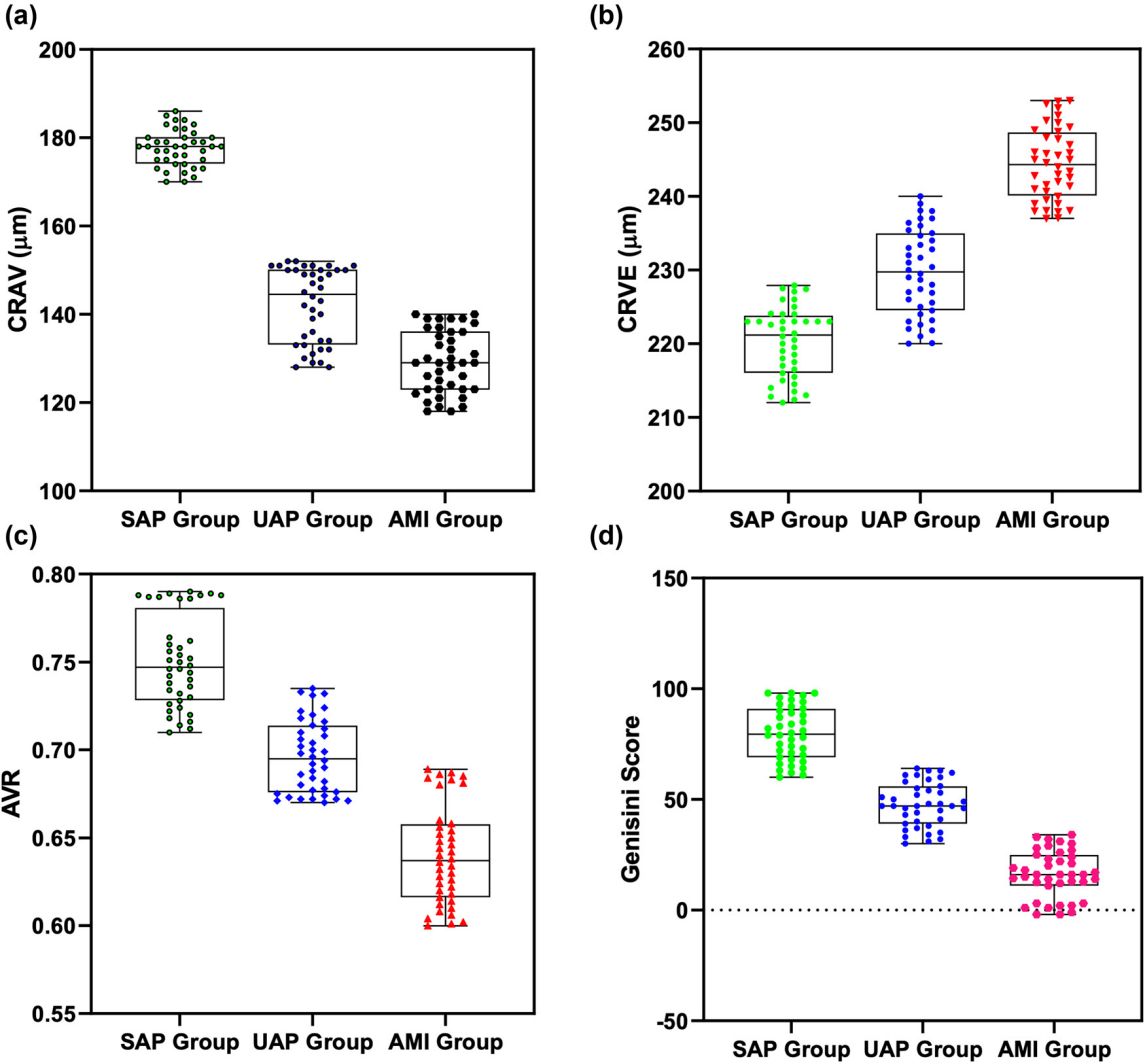
Comparison of CRAE, CRVE, AVR, and Gensini scores in different CHD groups. (a) CRAE in UA and AMI groups was reduced than that in the SAP group, while CRAE in the AMI group was reduced than that in the UA group. (b) CRVE of the UA group and the AMI group was enhanced than that of the SAP group, while CRVE of the AMI group was enhanced than that of the UA group. (c) AVR of the UA group and AMI group was reduced than that of the SAP group, while AVR of the AMI group was reduced than that of the UA group. (d) The Gensini score of the UA group and AMI group was enhanced than that of the SAP group, while the Gensini score of AMI group was enhanced than that of the UA group. Note: ***P < 0.001.

CRAE and AVR in UA and AMI groups were reduced compared to those in the SAP group (*P* < 0.05), while CRVE and Gensini scores in UA and AMI groups were enhanced compared to those in the SAP group (*P* < 0.05; Figure 2).

### 3.4 Comparison of CRAE, CRVE, AVR, and Gensini scores in different Gensini score groups

**Figure 3:**
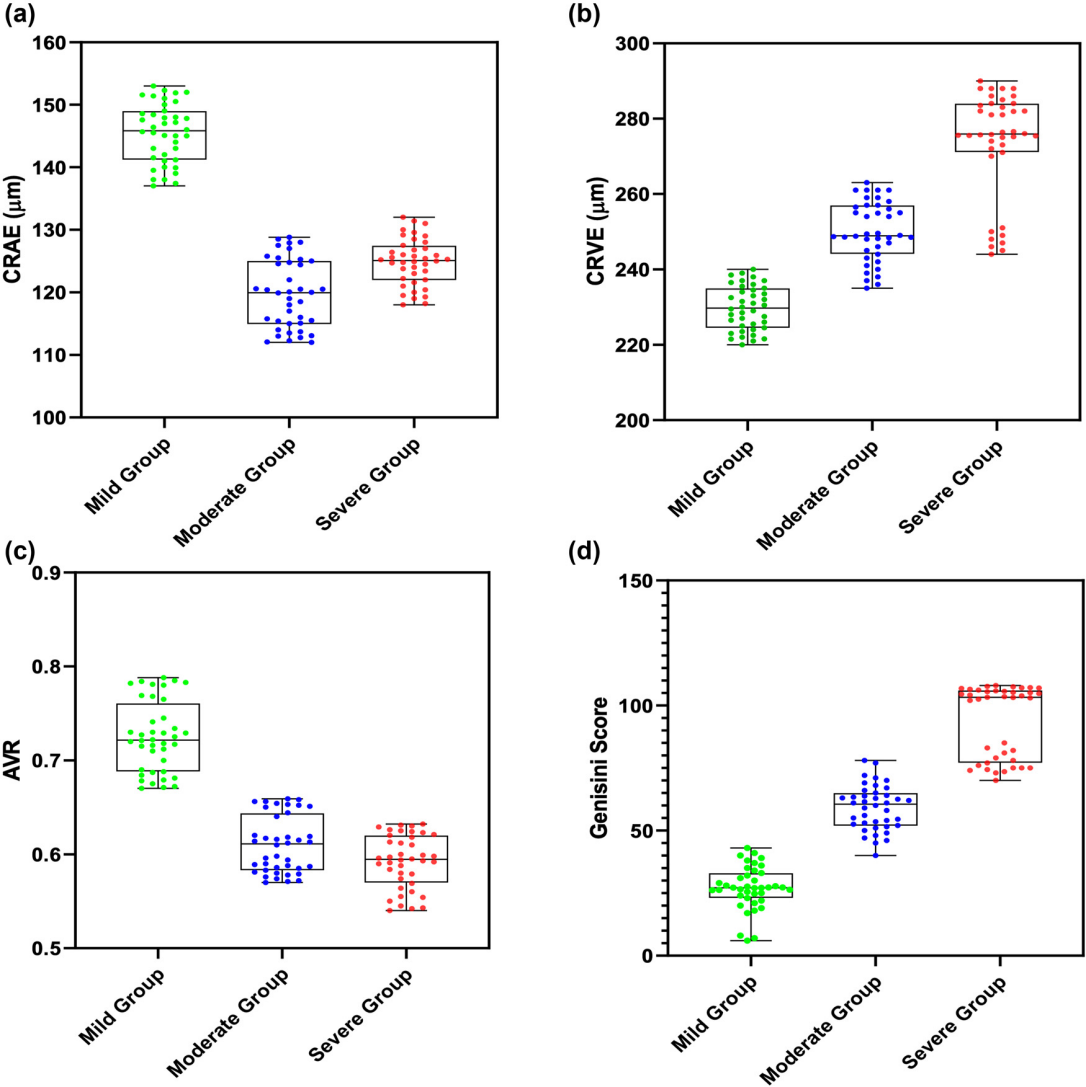
(a) CRAE of moderate and severe groups was reduced than that of the mild group, and CRAE of the severe group was reduced than that of the moderate group. (b) CRVE of moderate and severe groups was enhanced than that of the mild group, and CRVE of the severe group was enhanced than that of the moderate group. (c) AVR of the moderate and severe groups was reduced than that of the mild group, while AVR of the severe group was reduced than that of the moderate group. (d) Gensini scores in moderate and severe groups were enhanced than those in the mild group, and those in the severe group were enhanced than those in the moderate group. Note: ***P < 0.001.

CRAE and AVR in moderate and severe groups were reduced compared to those in the mild group (*P* < 0.05), while CRVE and Gensini scores in the severe group were enhanced compared to those in the mild group (*P* < 0.05; Figure 3).

### 3.5 Diagnostic value of AVR in patients with CHD

**Figure 4:**
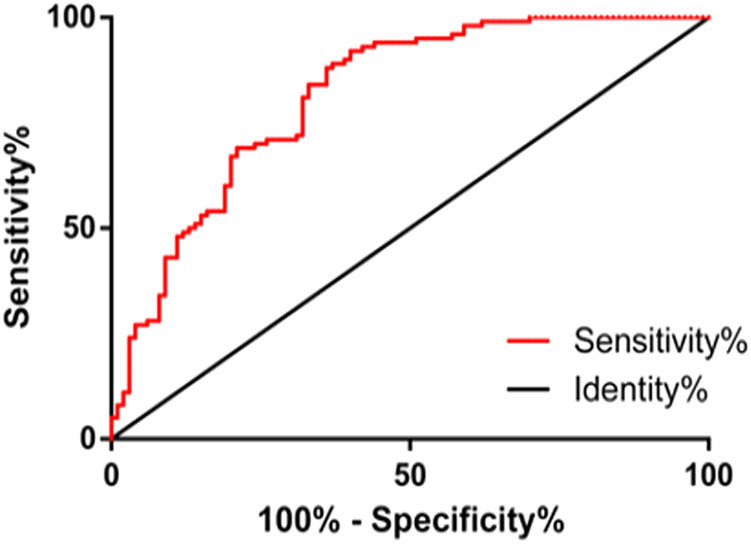
ROC curve of AVR diagnosing CHD. The sensitivity and specificity of AVR in diagnosing CHD were 92.00 and 60.00%, respectively.

By visualizing the ROC curve analysis, it was found that the AUC of AVR in diagnosing CHD was 0.815 (95% CI: 0.757–0.874), the cutoff value was 0.77, the diagnostic sensitivity was 92.00%, and the specificity was 60.00% (Table 2 and Figure 4).

**Table 2:** Diagnostic value of AVR in CHD patients

Indicators	AUC	95% CI	S.E	Cutoff	Sensitivity (%)	Specificity (%)
AVR	0.815	0.757–0.874	0.029	0.77	92.00	60.00

### 3.6 Correlation of CRAE, CRVE, and AVR with Gensini score

**Figure 5:**
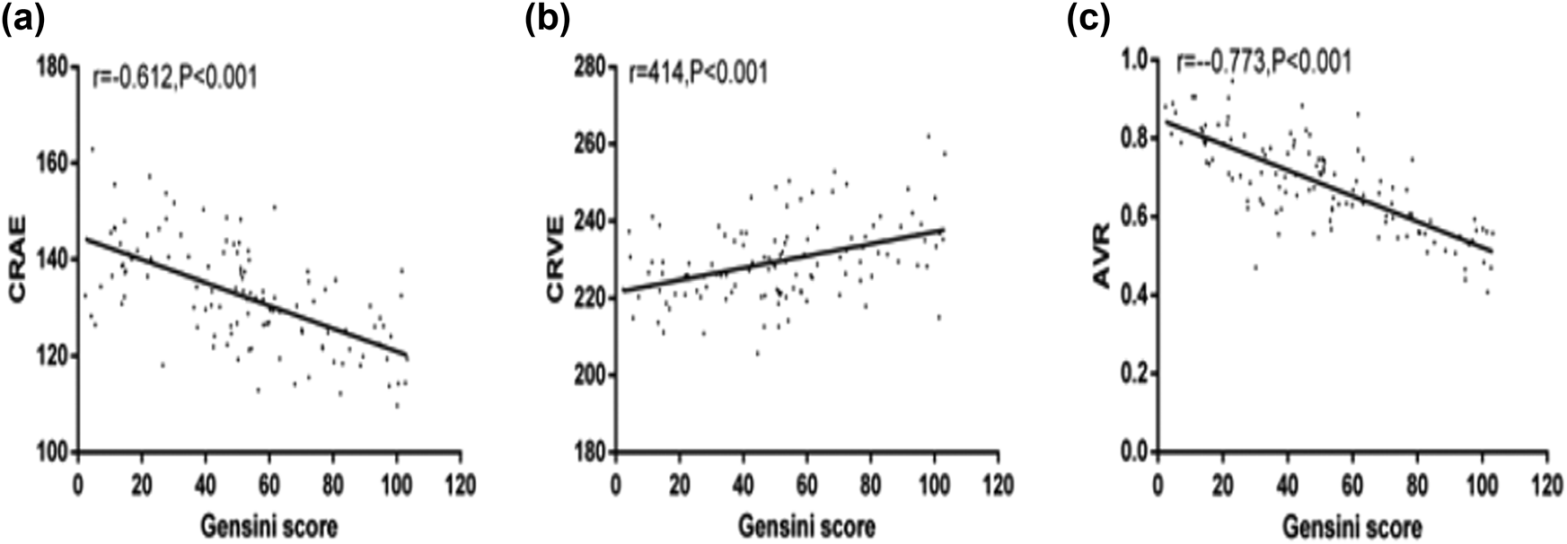
Correlation of CRAE, CRVE, and AVR with Gensini score. (a) CRAE was negatively correlated with Gensini score, *r* = −0.612, *P* < 0.001. (b) AVR was negatively correlated with Gensini score, *r* = −0.773, *P* < 0.001. (c) CRVE had a positive correlation with Gensini score, *r* = 0.414, *P* < 0.001.

Pearson correlation coefficient was applied to analyze the correlation of CRAE, CRVE, and AVR with Gensini score in CHD patients. The results showed that CRAE and AVR had a negative correlation with Gensini score (*r* = −0.612, *P* < 0.001; *r* = −0.773, *P* < 0.001) and CRVE had a positive correlation with Gensini score (*r* = 0.414, *P* < 0.001; Figure 5).

### 3.7 Risk factors of coronary stenosis in patients with CHD

The current study involves 40 CHD patients with Gensini score ≤30 as a good group and 80 CHD patients with Gensini score >30 as a bad group. After univariate analysis, the authors found that there was no evident difference in gender, average age, history of hypertension, drinking, TG, HDL-C, and LDL-C (*P* > 0.05), but there were evident differences in diabetes, smoking, and TC (*P* < 0.05; Table 3).

**Table 3:** Univariate analysis of coronary stenosis degree in CHD patients [*n* (%), mean ± SD]

Factor	*n*	Good group (*n* = 40)	Bad group (*n* = 80)	*χ*2/*t*	*P*
**Gender**				0.625	0.429
Male	72	22 (55.00)	50 (62.50)		
Female	48	18 (45.00)	30 (37.50)		
**Average age (years)**	120	63.68 ± 4.52	65.45 ± 4.92	1.720	0.089
**History of hypertension**				0.417	0.518
No	62	21 (52.50)	37 (46.25)		
Yes	58	19 (47.50)	43 (53.75)		
**History of diabetes**				6.158	0.013
No	88	35 (87.50)	53 (66.25)		
Yes	32	5 (12.50)	27 (33.75)		
**Drinking history**				0.582	0.445
No	92	29 (72.50)	63 (78.75)		
Yes	28	11 (27.50)	17 (21.25)		
**Smoking history**				4.838	0.027
No	64	27 (67.50)	37 (46.25)		
Yes	56	13 (32.50)	43 (53.75)		
**TC (mmol/L)**	120	4.12 ± 0.38	4.91 ± 0.50	8.121	0.001
**TG (mmol/L)**	120	1.33 ± 0.20	1.42 ± 0.28	1.686	0.095
**HDL-C (mmol/L)**	120	1.08 ± 0.14	1.14 ± 0.16	1.829	0.070
**LDL-C (mmol/L)**	120	2.97 ± 0.32	3.10 ± 0.37	1.722	0.088

Diabetes history, smoking history, and TC were included in the analysis, and they were assigned as independent variables. Taking the degree of coronary stenosis as the dependent variable, the logistic regression model was applied to carry out a multivariate analysis. The results showed that diabetes history, smoking history, and TC were independent risk factors for CHD patients (Tables 4 and 5).

**Table 4:** Logistic multivariate regression analysis assignment

Factor	Variable	Assignment
History of diabetes	X1	None = 0, yes = 1
History of smoking	X2	None = 0, yes = 1
TC	X3	The data belong to continuous variables and are analyzed with original data

**Table 5:** Multivariate analysis of coronary stenosis degree in CHD patients

Factor	*β*	SE	Wald	*P*	OR	95% CI
History of diabetes	1.968	0.624	14.529	<0.001	7.218	3.538–17.358
History of smoking	2.391	0.434	28.682	<0.001	10.127	5.357–23.505
TC	1.031	0.271	29.895	<0.001	3.845	2.062–7.140

## 4 Discussion

CHD refers to coronary artery disease syndrome caused by the insufficient blood supply to the heart and coronary artery stenosis [22]. The morbidity and fatal disability rates are very high, causing great harm to patients’ lives and health, and it has become a common public health problem of fatal diseases [23]. Therefore, it is of great significance to detect and monitor the progress of the disease in time and judge the severity of the lesions for the prevention and treatment of CHD [24,25]. This study predicted and tested the severity of the lesions in CHD patients by quantitatively measuring the diameter of retinal blood vessels.

Previous research has shown that the change of retinal vessel diameter is related to hypertension, diabetes, and cerebrovascular diseases and has a particular suggestive effect on CHD [26,27]. However, few reports are available on the correlation of retinal vessel diameter with CHD. The results showed that CRAE and AVR of CHD patients were lower than those of normal people, while CRVE was higher than that of normal people, indicating that the retinal artery of senile CHD patients was narrowed, the vein was dilated, and the ratio of artery and vein became smaller. Chandra et al. [28] revealed that retinal artery stenosis and vein dilatation were related to atherosclerotic heart failure, and the risk of occurrence could be predicted. In the study by Anyfanti et al. [29], it was reported that the retinal artery of rheumatoid arthritis patients was smaller than that of the healthy people, which was related to inflammatory reaction and could play a predictive role in cardiovascular disease. The authors further analyzed CRAE, CRVE, AVR, and Gensini scores of patients with CHD in different clinical types and pathological degrees. The results showed that CRAE and AVR scores of patients with UA and AMI were smaller than those of SAP, while CRVE and Gensini scores were higher than those of SAP. CRAE and AVR of severe and mild patients were lower than those of mild patients, and CRVE and Gensini scores were higher than those of mild patients [30]. With the severity of CHD, CRVE and Gensini scores were reduced, while CRVE and Gensini scores were enhanced. The correlation of CRAE, CRVE, and AVR with Gensini score was analyzed by the Pearson correlation coefficient. The results show that CRAE and AVR had a negative correlation with the Gensini score, while CRVE was positively correlated with the Gensini score, indicating that the low CRAE and AVR were related to the high CRVE. That suggested that with the deterioration of CHD, the comprehensive index of AVR was more correlated with the Gensini score. In the research of Kim et al. [31], it was suggested that compared with the control group, patients with retinal artery occlusion had an enhanced prevalence of subclinical coronary artery diseases and more extensive coronary artery lesions. Also, in the study by Wang et al. [32], retinal blood vessels were related to CAD degree and Gensini score, which was similar to our research results. The study of the retinal vascular system in senile dementia found a certain correlation between retinal vascular disease and cerebrovascular disease, and retinal vascular characteristics can be applied as a noninvasive index for diagnosis and prognosis of dementia patients [33]. As a comprehensive evaluation index of retinal vascular disease, AVR not only reflects the reduction of retinal arterioles but also reflects the expansion of retinal venules. The decrease of AVR is an important sign of retinal arteriosclerosis and vascular stenosis. Therefore, in this study, the authors visualized the ROC curve of AVR in diagnosing CHD patients and concluded that the AUC of AVR in diagnosing CHD patients was 0.815, which indicated that AVR has a great value in diagnosing CHD patients and was expected to become a clinical diagnostic index. Finally, the authors also analyzed the risk factors of coronary artery disease in CHD patients. The results showed that diabetes history, smoking history, and TC were independent risk factors. According to Gururani et al. [34], the risk factors of coronary artery disease in patients with CHD were diabetes and testosterone level, and both TC and LDL were risk factors, which were the same as the results of this study.

The authors preliminarily discussed the correlation of CRAE, CRVE, and AVR with Gensini score and predicted that AVR might be applied as a noninvasive marker for CHD diagnosis, which provided a clinical basis for its prognosis. However, there is still room for improvement in this study. For example, the relationship between the dynamic changes of retinal blood vessels and coronary artery disease can be observed for a long time, and this needs to be followed up for some time to strengthen the credibility. In the future, the aforementioned directions will be used for further improvement.

## 5 Conclusion

To sum up, CRA and AVR are negatively correlated with the Gensini score, while CRVE is positively correlated with the Gensini score. Therefore, AVR is expected to be a noninvasive index to diagnose and predict senile CHD, which has a particular evaluation value. In addition, diabetes, smoking history, and TC are independent risk factors of senile CHD.
